# Characterization of Brain Volume Changes in Aging Individuals With Normal Cognition Using Serial Magnetic Resonance Imaging

**DOI:** 10.1001/jamanetworkopen.2023.18153

**Published:** 2023-06-28

**Authors:** Shohei Fujita, Susumu Mori, Kengo Onda, Shouhei Hanaoka, Yukihiro Nomura, Takahiro Nakao, Takeharu Yoshikawa, Hidemasa Takao, Naoto Hayashi, Osamu Abe

**Affiliations:** 1 Department of Radiology, The University of Tokyo, Bunkyo, Tokyo, Japan; 2 Department of Radiology, Juntendo University, Bunkyo, Tokyo, Japan; 3 Russell H. Morgan Department of Radiology and Radiological Science, Johns Hopkins University School of Medicine, Baltimore, Maryland; 4 F.M. Kirby Research Center for Functional Brain Imaging, Kennedy Krieger Institute, Baltimore, Maryland; 5 Department of Computational Diagnostic Radiology and Preventive Medicine, The University of Tokyo Hospital, Bunkyo, Tokyo, Japan; 6 Center for Frontier Medical Engineering, Chiba University, Inage, Chiba, Japan

## Abstract

**Importance:**

Characterizing longitudinal patterns of regional brain volume changes in a population with normal cognition at the individual level could improve understanding of the brain aging process and may aid in the prevention of age-related neurodegenerative diseases.

**Objective:**

To investigate age-related trajectories of the volumes and volume change rates of brain structures in participants without dementia.

**Design, Setting, and Participants:**

This cohort study was conducted from November 1, 2006, to April 30, 2021, at a single academic health-checkup center among 653 individuals who participated in a health screening program with more than 10 years of serial visits.

**Exposure:**

Serial magnetic resonance imaging, Mini-Mental State Examination, health checkup.

**Main Outcomes and Measures:**

Volumes and volume change rates across brain tissue types and regions.

**Results:**

The study sample included 653 healthy control individuals (mean [SD] age at baseline, 55.1 [9.3] years; median age, 55 years [IQR, 47-62 years]; 447 men [69%]), who were followed up annually for up to 15 years (mean [SD], 11.5 [1.8] years; mean [SD] number of scans, 12.1 [1.9]; total visits, 7915). Each brain structure showed characteristic age-dependent volume and atrophy change rates. In particular, the cortical gray matter showed a consistent pattern of volume loss in each brain lobe with aging. The white matter showed an age-related decrease in volume and an accelerated atrophy rate (regression coefficient, −0.016 [95% CI, −0.012 to –0.011]; *P* < .001). An accelerated age-related volume increase in the cerebrospinal fluid–filled spaces, particularly in the inferior lateral ventricle and the Sylvian fissure, was also observed (ventricle regression coefficient, 0.042 [95% CI, 0.037-0.047]; *P* < .001; sulcus regression coefficient, 0.021 [95% CI, 0.018-0.023]; *P* < .001). The temporal lobe atrophy rate accelerated from approximately 70 years of age, preceded by acceleration of atrophy in the hippocampus and amygdala.

**Conclusions and Relevance:**

In this cohort study of adults without dementia, age-dependent brain structure volumes and volume change rates in various brain structures were characterized using serial magnetic resonance imaging scans. These findings clarified the normal distributions in the aging brain, which are essential for understanding the process of age-related neurodegenerative diseases.

## Key Points

**Question:**

What are the spatial profiles of longitudinal changes in brain volume and their rates in aging individuals with normal cognition, at the population and individual levels?

**Findings:**

In this cohort study, 653 adults with normal cognition were followed up annually for over 10 years, with a total of 7915 visits. The volume of each brain structure exhibited characteristic age-related trajectories, with acceleration of volume reduction over age and cross-individual variability noted in individuals with normal cognition.

**Meaning:**

These results provide insight into brain aging at the individual level, which is essential for understanding the process of age-related neurodegenerative diseases.

## Teaser

This cohort study investigates age-related trajectories of the volumes and volume change rates of brain structures in participants without dementia.

## Introduction

In recent years, a consensus has been reached that various neurodegenerative diseases begin decades before symptomatic onset.^1,2,3^ However, previous investigations of brain aging over the sensitive time period from middle to old age have been limited by cross-sectional designs or small sample sizes. A recent meta-analysis based on more than 100 publications (>120 000 brain magnetic resonance imaging [MRI] scans) revealed a paucity of data, particularly for individuals approximately 45 years of age, with fewer than 500 individuals of this age available collectively across all publications.^4^ Furthermore, limited longitudinal data are available. To our knowledge, no large-scale (>100 participants) longitudinal study in which participants were followed up annually for more than 10 years is available. Therefore, our understanding of the aging process is limited, which prevents characterization of the development of pathologic conditions in their early stages.

Magnetic resonance imaging allows for the noninvasive observation of brain anatomy and is suitable for studying brain aging processes. Numerous cross-sectional MRI studies have reported brain atrophy to be associated with normal aging, with generally comparable results,^5,6,7,8^ including findings of volume loss in the frontal lobe,^7^ temporal lobe,^9^ and hippocampus^9,10^ and increases in cerebrospinal fluid (CSF) spaces.^11,12^ These changes do not occur uniformly across all the brain regions.^13,14^ An association between volume loss and brain function has also been reported.^15,16^

Although cross-sectional studies have provided valuable insights into age-related changes in the brain, they have limitations.^17^ Interindividual differences in brain size and age-related changes reduce the sensitivity of these studies in characterizing the aging process and the differences between groups. Furthermore, these studies do not allow us to measure variability in the rate of atrophy because they rely on the assumption that brain aging processes are invariant across individuals.

Longitudinal studies can avoid many of the limitations of such cross-sectional studies by tracking changes in the brain structure over time at the individual level, which allows for the separation of interindividual and intraindividual variations, providing more precise estimates of atrophy rates. Previous longitudinal studies have shown that brain atrophy begins at an age in the 40s and that the annual volume change rate increases with age.^18,19,20,21,22,23,24^ However, most of these studies were based on small sample sizes (typically <100 individuals) and had short follow-up periods (typically 1-5 years), limiting their ability to accurately estimate the mean (SD) values of changes and the associations among different brain structures. Studies with longer follow-up periods are needed to provide more precise estimates of the various brain structures. Moreover, long-term follow-up studies have the potential to provide data about the progression of neurodegenerative diseases from their prodromal phases, which is key to understanding their pathologic characteristics and for developing preventive measures.

In this study, we used large-scale data from brain MRI scans in Japan, which had been recorded as part of an annual health checkup system called Brain Dock.^12^ Based on these data, we aimed to assess changes in brain structural volume and atrophy rates over time in individuals without dementia. We included longitudinal annual follow-up data of approximately 8000 visits to a single institute to evaluate the association of normal aging with regional brain volumes and atrophy rates. We hypothesized that there would be individual differences in the degree and rate of atrophy in individuals with normal cognition, which could not be measured by cross-sectional studies. We aimed to understand how the aging process is associated with different regions of the brain by characterizing age-related structural changes across tissue types (gray matter [GM], white matter [WM], and CSF) and regions (whole brain, major brain lobes, and structures known to be associated with dementia). We also evaluated intraindividual and interindividual differences in age-related changes in brain structures.

## Methods

### Study Population and Sample

This single-site prospective study was approved by the institutional review board of The University of Tokyo Hospital, Tokyo, Japan. This institution conducts a comprehensive health screening program, providing annual checkups for participants over the long term. Before participating in the program, written informed consent was obtained from all participants for use of their clinical, laboratory, and imaging data for research purposes. We followed the Strengthening the Reporting of Observational Studies in Epidemiology (STROBE) reporting guideline.

Of the 10 209 participants who participated in the comprehensive health screening program from November 1, 2006, to April 30, 2021, those who underwent more than 10 annual checkups were included in this study (eFigure 1 in Supplement 1). Clinical history was recorded at each visit, and the presence of cognitive impairment was reported by an internal medicine physician. In detail, the participant first fills out a questionnaire, which is then reviewed by a nurse and finally confirmed by an internal medicine physician who conducts the examination and final confirmation. An objective cognitive function evaluation was conducted at every visit using the Mini-Mental State Examination and the Wechsler Memory Scale–Revised. The exclusion criteria were a Mini-Mental State Examination score of 24 or lower during the follow-up period, contraindications to MRI, a history of neurologic or psychiatric disorders, and major intracranial findings detected on MRI scans, including intracranial hemorrhage, infarction, and masses.

### Brain Imaging

All participants underwent high-resolution, 3-dimensional (3D) T1-weighted volumetric MRI scans on a 3.0-T system, either Signa EXCITE (GE HealthCare), Discovery MR750 (GE HealthCare), or Biograph mMR (Siemens Healthcare GmbH). Because we aimed to depict age-related changes longitudinally, we kept the image acquisition protocols, scanner hardware, and software versions as uniform as possible throughout the study. Details of the acquisition parameters are provided in the eMethods in Supplement 1.

For the examination of intracranial findings, T2-weighted images, T2-weighted fluid-attenuated inversion recovery images, and diffusion data were obtained. Image inspections were performed by an MR operator during the scans. After the images were transferred to the picture archiving and communication system, all images were independently examined by 2 radiologists for gross structural abnormalities and major intracranial findings.

### Brain Segmentation

Automated whole-brain segmentation was performed on high-resolution 3D T1-weighted images using a multiatlas pipeline implemented in MVision (Corporate M) and derived from MRICloud (Johns Hopkins University). This method segments whole-brain MRI data into multiple hierarchical structural levels.^25,26^ Each level contained a set of predefined regions of interest, and the volumes of each of these regions were measured.^26,27^ The test-retest reproducibility of T1-volumetric analysis has been reported to be extremely high.^28^

Brain segmentation was performed by an operator who was blinded to participants’ clinical demographic information. For the purposes of this study, we evaluated the whole brain, brain lobes (frontal, parietal, occipital, temporal, limbic, and cerebellar), cortical GM, subcortical GM, WM, ventricles, sulci, hippocampus, and amygdala. We examined tissue type (GM, WM, and CSF) and region (frontal, parietal, temporal, occipital, limbic, cerebellum, hippocampus, and amygdala) in separate analyses. The analysis of lobar volumes included only the cortical GM.

### Brain Structure Volumes

The imaging protocol was subject to software upgrades and scanner replacement during the study period. Differences caused by different imaging protocols were evaluated and calibrated based on regression analysis. Multivariate linear regression was performed to investigate the association of age (as a continuous covariate) and protocol (as a factor covariate; 0 or 1) with the volume changes for each brain region. This approach assumes that a linear association exists between brain structure volume (dependent variable) and age (independent variable). To confirm the effect of calibration, the coefficient of determination (*R^2^*) of the regression by age was compared before and after protocol calibration. To enable comparisons between individuals with different head sizes, the calibrated absolute volume for each region of interest was converted into relative volumes by dividing the raw volume of each given structure by the total volume, which was calculated by summing all structures.

### Brain Structure Volume Change Rate

To calculate the annual rates of change for each brain structure, we used a regression model to assess the association of the volume of the structure (dependent variable) with the age of the participant (independent variable). The volume of the structure used here was not normalized to the total structure volume because we sought to measure within-individual longitudinal changes, which are not correlated with the total structure volume for these measures. This allowed us to estimate within-individual annual tissue loss for each structure. To ensure comparability of the calculated volume change rates across different brain structures, we normalized the absolute volume loss for each structure by dividing it by the mean value of all data points for that structure. We then repeated the regression analysis using the normalized volume loss as the dependent variable and age as the independent variable. This allowed us to estimate the annual rate of change in volume for each structure based on the slope of the regression line.

### Statistical Analysis

R, version 3.3.0 (R Group for Statistical Computing) and Python, version 3.7 (Python Foundation) were used for all statistical analyses. All *P* values were from 2-sided tests and results were deemed statistically significant at *P* < .05. The absolute volumes of left- and right-sided structures were combined. We plotted the brain structure volume, normalized for head size, against age. The distribution of brain structure volume and age-related volume changes was displayed for different age groups. The generalized additive model included a smooth term for age, and the default cubic splines were used as the base function to fit the model.^29^ The identity link function was used for the generalized additive model. Only age was adjusted during model fitting.

## Results

Overall, 653 participants, aged 33 to 95 years (mean [SD] age at baseline, 55.1 [9.3] years; median age at baseline, 55 years [IQR, 47-62 years]; 447 men [69%] and 206 women [32%]), were evaluated annually for up to 15 years (mean [SD], 11.5 [1.8] years; mean [SD] number of scans, 12.1 [1.9]; total visits, 7915). The participants’ background characteristics are listed in the Table. The sex distribution across different age categories in the cohort is described in eTable 1 in Supplement 2. The coefficient of the determinations (*R*^2^) of the age dependency of brain structure volume increased with protocol calibration (cortical GM, from 0.119 to 0.140; subcortical GM, from 0.08 to 0.09; WM, from 0.112 to 0.133; and cerebellum, from 0.129 to 0.152).

**Table.** Characteristics of the Study Sample

Characteristic	Participants (N = 653)
Age at baseline, y	
Mean (SD)	55.1 (9.3)
Median (IQR)	55 (47-62)
Follow-up period, y	
Mean (SD)	11.5 (1.8)
Median (IQR)	11.5 (10.2-13.1)
Sex, No. (%)	
Women	206 (32)
Men	447 (69)
Mini-Mental State Examination score	
Mean (SD)	29.4 (0.9)
Median (IQR)	30 (29-30)
Wechsler Memory Scale–Revised score	
Mean (SD)	33.8 (6.5)
Median (IQR)	34 (30-38)
Age group in years, No./total No. (%) of visits	
30s	36/7915 (0.5)
40s	1269/7915 (16)
50s	2624/7915 (33)
60s	2553/7915 (32)
70s	1256/7915 (16)
80s	169/7915 (2)
90s	8/7915 (0.1)
No. of visits, No. (%) of participants	
10	192 (29)
11	86 (13)
12	123 (19)
13	84 (13)
14	76 (12)
15	55 (8)
16	37 (6)
Total No. of visits	7915

### Age-Dependent Brain Volume Change

**Figure 1.**
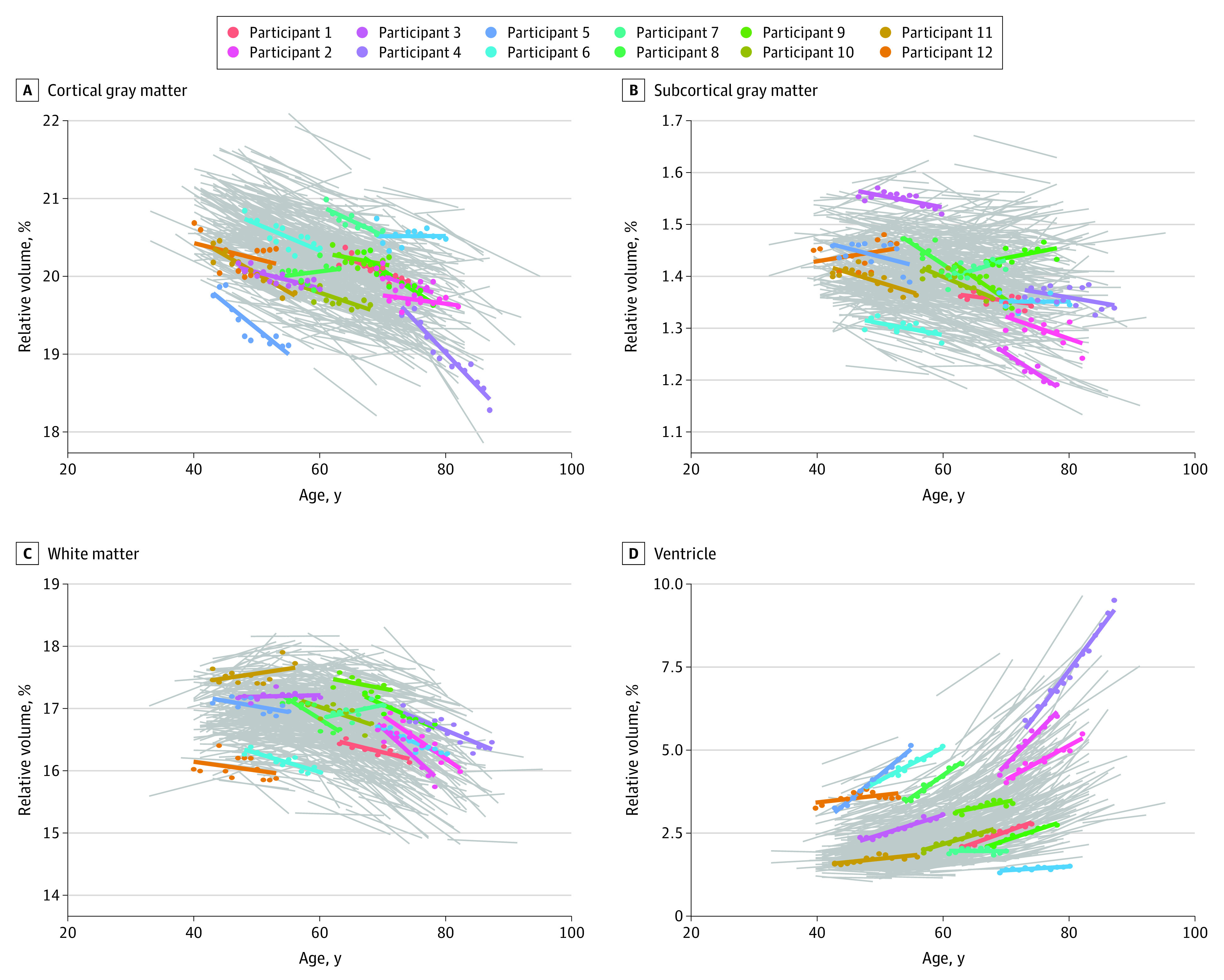
Longitudinal Changes in Brain Structure Volumes Over Time in Individual Participants Each line (gray and colored) indicates 10 to 15 years of change for a single participant. Representative cases are color coded. The y-axis shows the normalized volume of the brain structure as a percentage of the total volume of all structures combined.

Figure 1 shows the longitudinal change in the brain structure volume over time. A volume decrease in the brain parenchyma and an increase in the CSF space (ventricle and sulcus) were observed; overall, an annual decrease of –0.4% in whole-brain volume was observed, with a decrease rate ranging from –0.3% per year among individuals in their 40s to –0.5% per year among individuals in their 80s. There was an overall annual increase in ventricle volume of 1.8%, with a range of 1.0% per year among individuals in their 40s to 2.5% per year among individuals in their 80s. Interindividual differences were present in the trends of volume changes in brain structures during follow-up, even among individuals in the same age range. eFigure 2 in Supplement 1 shows the volume distribution of the population across different ages. The volume distribution was wider in the older age groups.

Several notable features of aging-related changes in brain volume were observed. First, the whole-brain volume showed accelerated volume loss and exhibited wider volume distribution as age increased; an annual decrease of –0.4% in whole-brain volume was observed, with a decrease rate ranging from –0.3% per year among individuals in their 40s to –0.5% per year among individuals in their 80s. Second, within the cortical GM, the frontal and temporal lobes showed a large age dependency (annual volume decrease, –0.4% and –0.5%, respectively), whereas the parietal and occipital lobes and limbic system showed a small age dependency (annual volume decrease, –0.3%, –0.3%, and –0.3%, respectively). Third, regional differences in age-related WM volumes were observed. Considerable age-related changes were observed in the anterior and inferior WM (annual volume decrease, –0.5% and –0.5%, respectively). The posterior WM showed a small age dependency (annual volume decrease, –0.3%). Fourth, the ventricular and sulcus volumes showed marked age-dependent changes (overall annual increase, 1.8% for ventricle volume and 0.5% for sulcus volume).

### Longitudinal Associations Between Brain Structure Volumes

**Figure 2.**
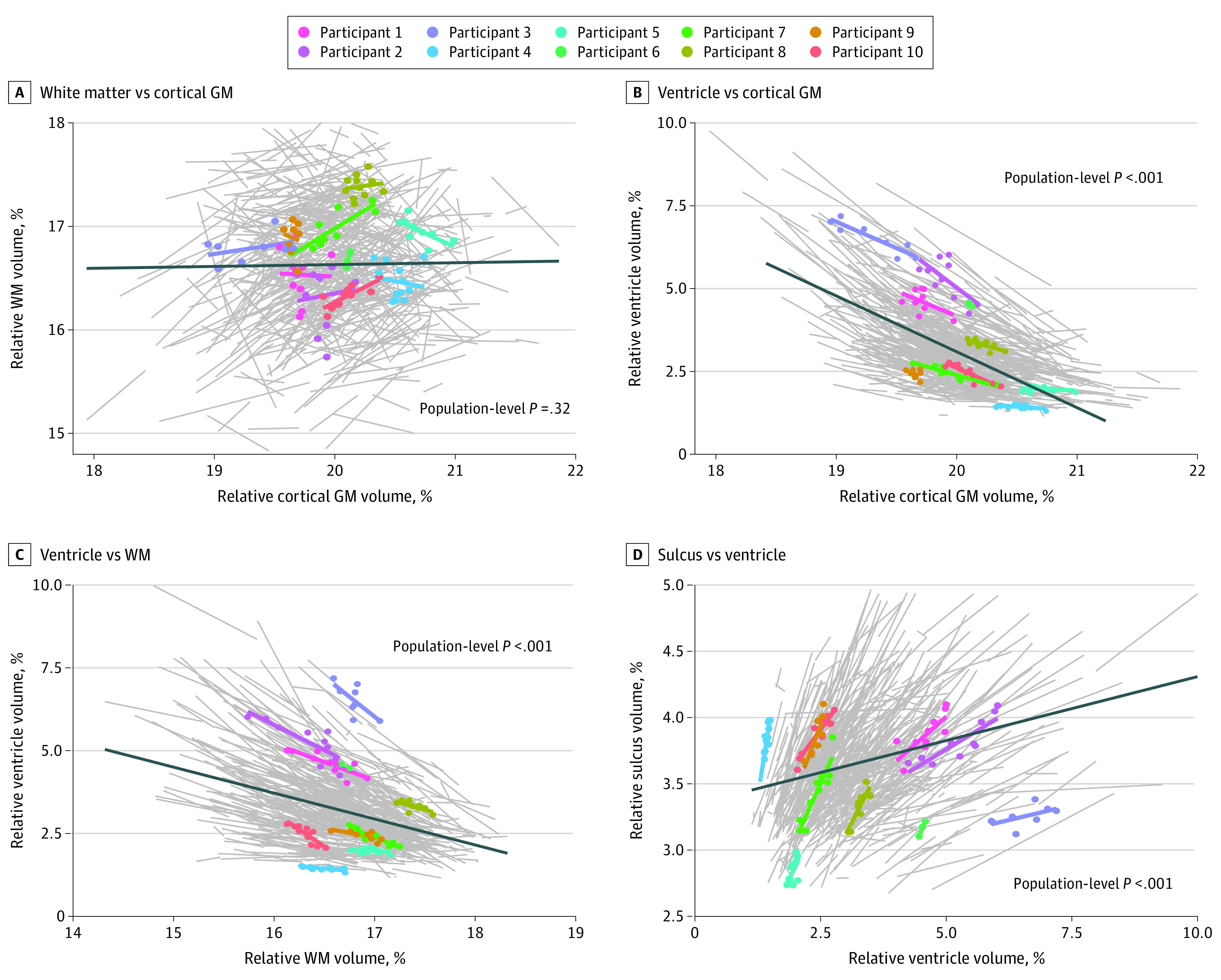
Longitudinal Association Between Brain Structure Volumes Over Time Within Different Tissue Types The association between brain structure volumes over time within different tissue types is shown. Best-fit lines for each individual (gray lines, individual data), with representative cases visualized using sample points, are color coded. The dark blue solid line is the linear fit using data from all visits of all participants (cross-sectional population data). GW indicates gray matter; WM, white matter.

Figure 2 and eFigure 3 in Supplement 1 show the longitudinal association between brain structure volumes over time. There were structural combinations for which correlations were weak at the population level but for which strong correlations were obtained when analyzed longitudinally at the individual level (eTable 2 in Supplement 1), such as the WM-ventricle combination (group level, *R*^2^ = 0.158; individual level, *R*^2^ = 0.588). The slopes of the correlations also showed large individual differences. Ventricle-sulci and WM-sulci combinations showed linear associations at both individual and group levels (volume-change *R*^2^ = 0.0855 at the group level and *R*^2^ = 0.736 [95% CI, 0.451-0.868] at the individual level for ventricle-sulci, and volume-change *R*^2^ = 0.4590 at the group level and *R*^2^ = 0.658 [95% CI, 0.319-0.855] at the individual level for WM-sulci). The cerebral cortex-WM combination showed a weak correlation at both the individual and group levels (volume-change *R*^2^ = 0.0002 at the group level and *R*^2^ = 0.260 [95% CI, 0.072-0.519] for individual level).

### Longitudinal Brain Volume Change Rate

**Figure 3.**
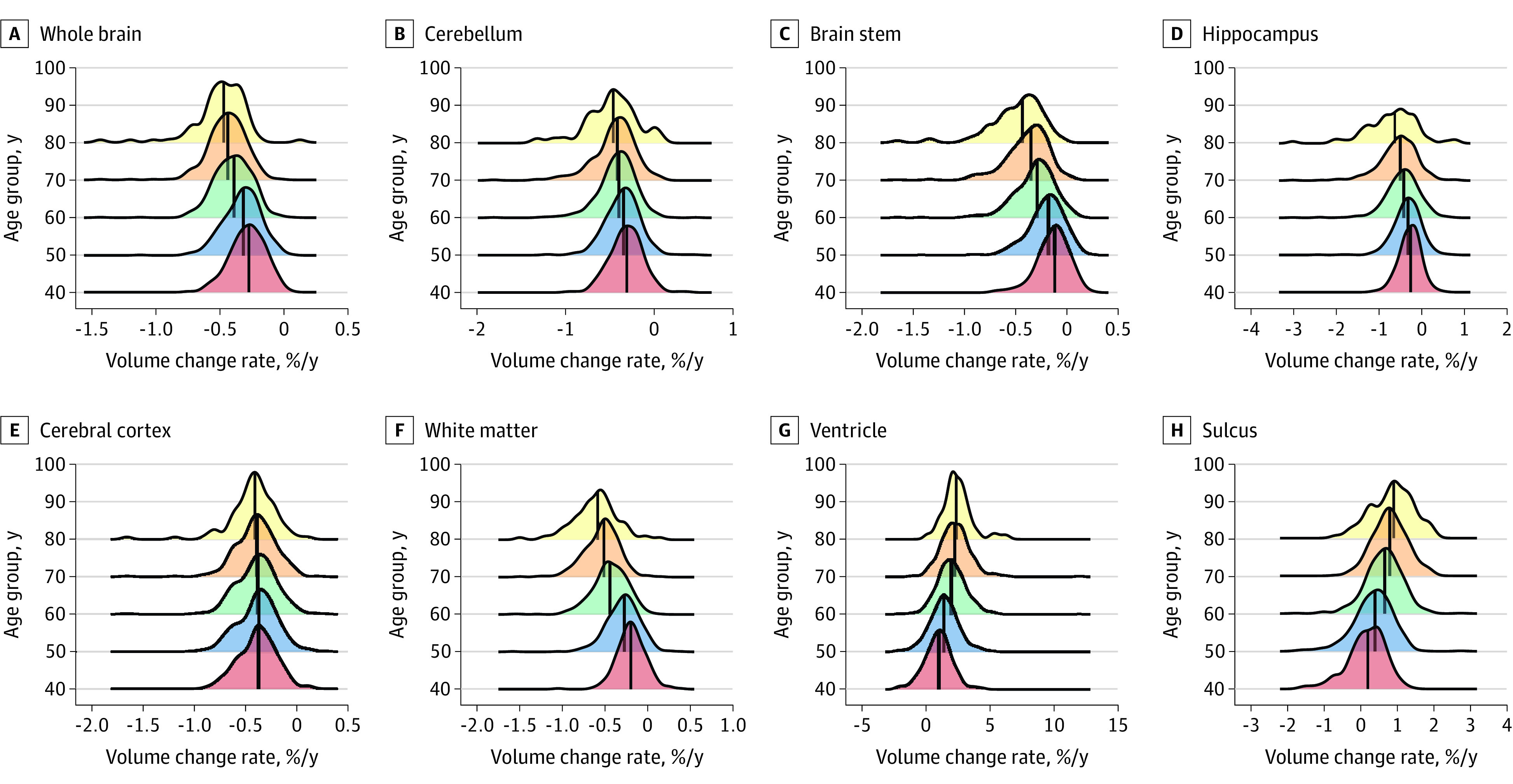
Ridgeline Plots of the Distribution of Brain Structure Volume Change Rates for Each Decade Solid black lines indicate the median values. Different colors indicate different age groups. The x-axis shows the normalized volume change per year as a percentage of the absolute volume of the given structure.

Figure 3 shows change rates in brain volumes in the study population across different age groups. Detailed results for each brain structure are presented in eFigure 4 and eTable 3 in Supplement 1. The acceleration of whole-brain volume change rates was confirmed. Each brain lobe, other than the temporal lobe, exhibited a relatively stable or constant volume change rate, regardless of age group (this rate was smaller in the occipital and limbic regions) (eFigure 4 in Supplement 1). In contrast to the cortical GM, WM exhibited an acceleration in the volume change rate (regression coefficient, −0.016 [95% CI, −0.012 to –0.011]; *P* < .001). All WM regions, including the posterior WM, showed accelerated volume change rate with age. Although the absolute value of the rate of volume change in the occipital WM was small, the rate of volume change increased with age. Ventricular and sulcus volumes showed accelerated volume change rates and a wider distribution of volume change rates (ventricle regression coefficient, 0.042 [95% CI, 0.037-0.047]; *P* < .001; sulcus regression coefficient, 0.021 [95% CI, 0.018-0.023]; *P* < .001) in the older age groups. Sex-stratified analyses of change rates in brain volumes are reported in eTables 4 and 5 in Supplement 1.

**Figure 4.**
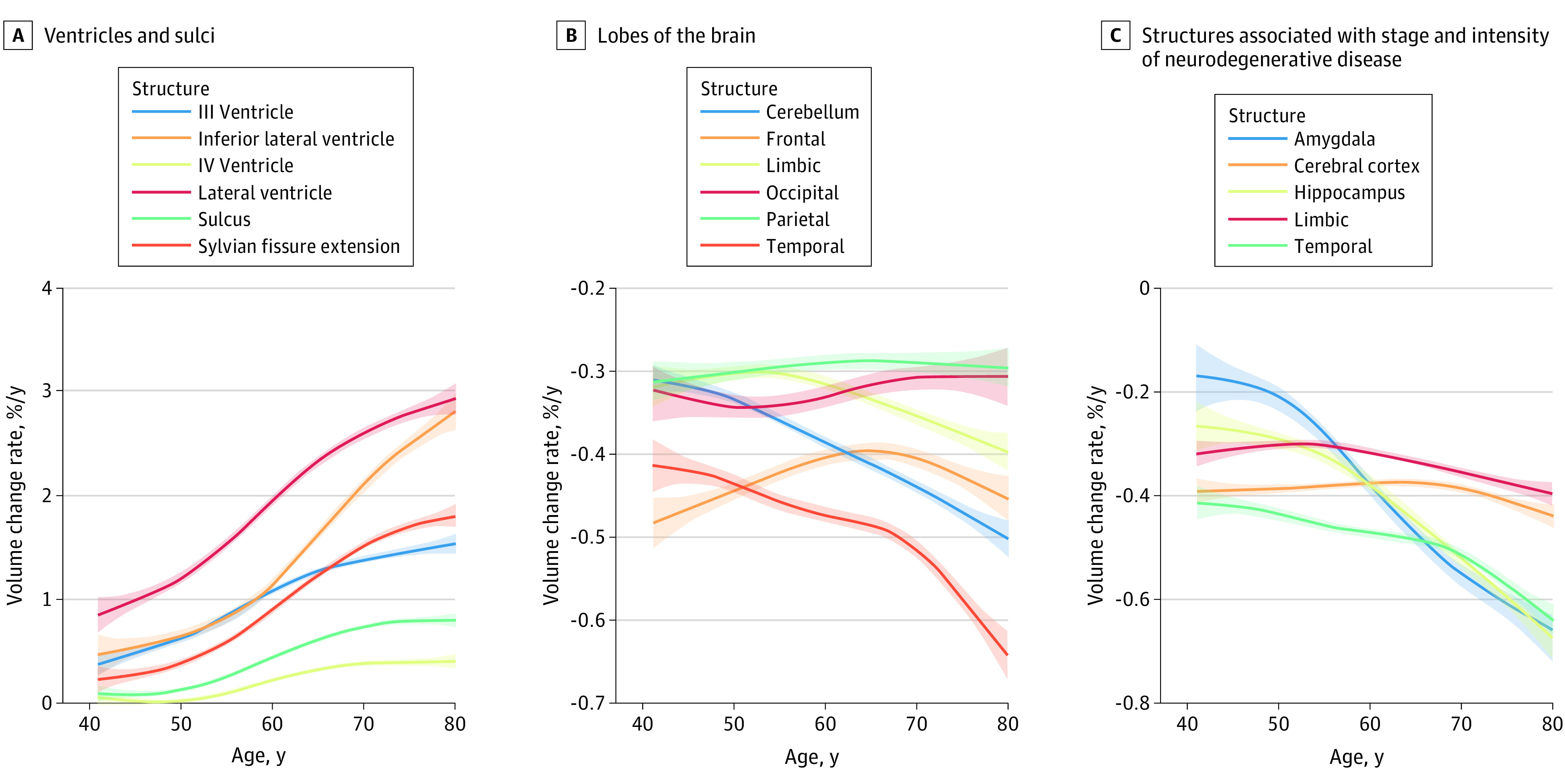
Visualization of Age-Dependent Volume Change Rate for Each Tissue Type For each structure, age-dependent changes in the volume change rate were fitted using a generalized additive model with cubic splines as a base function. The shaded areas indicate the 95% CIs.

Figure 4 shows the time course of the brain structure volume change rate over time in the study population. Specific patterns of change were observed within this overall acceleration. The volume change rate of the lateral ventricle exhibited the largest acceleration with aging, reaching a volume loss of up to 3% per year for those in their 80s (annual volume increase, 2.5%). The rate of volume increase also increased in the Sylvian fissure starting in the 50s. The volume change rate of the cerebellum increased linearly with age (annual volume decrease, –0.3%, –0.4%, –0.4%, –0.5%, and –0.5% among people in their 40s, 50s, 60s, 70s, and 80s, respectively). The volume change rate of the temporal lobe also accelerated with age, particularly from the 70s. The volume change rate acceleration with age in the cerebral cortex was relatively gradual (annual volume decrease, –0.4%, –0.4%, –0.4%, –0.4%, and –0.4% among individuals in their 40s, 50s, 60s, 70s, and 80s, respectively), whereas that in the hippocampus (annual volume decrease, –0.3%, –0.3%, –0.5%, –0.6%, and –0.7% among individuals in their 40s, 50s, 60s, 70s, and 80s, respectively) and temporal lobe (annual volume decrease, –0.4%, –0.5%, –0.5%, –0.5%, and –0.6% among individuals in their 40s, 50s, 60s, 70s, and 80s, respectively) was rapid. Acceleration of the volume change rate of the hippocampus preceded that of the temporal lobe. The change rates of the hippocampus and amygdala were slower than that of the entire cerebral cortex before 60 years of age, indicating that they were relatively preserved in the earlier stages of life.

## Discussion

In this large-scale prospective cohort study, we followed up with a sample of adults without dementia using serial MRI scans over the long term to provide spatial and temporal profiles of age-dependent changes in the brain without known neurologic conditions. To our knowledge, this study included the largest number of participants (n = 653) who underwent annual brain MRI examinations for a period exceeding 10 years, making it the most detailed longitudinal follow-up study of brain structure changes in normal aging. This allowed us to characterize atrophy rates and their changes (acceleration) with age and to reveal population variability in atrophy rates. Our findings revealed considerable individual variability in the rates of decrease, even in healthy middle-aged individuals. Defining the pattern and rate of these changes is crucial to understanding the long-term processes of various neurodegenerative disorders.

The cortical GM showed a characteristic pattern of volume loss in each brain lobe with age; the frontal, parietal, and temporal lobes showed the greatest decrease. This regional volume loss pattern was consistent with that noted in previous studies.^20^ A 2-phase change in the rate of volume loss was observed in the frontal lobe (Figure 4). This finding was consistent with the results of a previous longitudinal study.^30^ The first phase involved rapid acceleration of volume loss before the age of 50 years, followed by a period of relatively lower volume loss in the 60s, after which the rate of volume loss accelerated again. The volume change rate of the temporal lobe accelerated starting at approximately 70 years of age. This acceleration was preceded by an increase in the volume change rate in the hippocampus and amygdala. Although a previous study by Scahill et al^20^ also showed this tendency, their study was limited to a small number of brain structures (whole brain, temporal lobe, hippocampus, and ventricle) with a limited number of participants (n = 39; age range, 31-84 years) with only 1 follow-up examination after a 1-year interval. Our results also showed that the volume loss of the cortical GM begins gradually and progresses slowly, whereas WM loss starts later and progresses more rapidly, with age-associated acceleration. This finding is consistent with those of previous studies.^4,31^

Although the overall spatial and temporal profiles of age-related atrophy agreed with those obtained in previous studies, we uniquely characterized individual-level atrophy rates, acceleration rates, and their cross-individual variability in the present study (eFigure 2 in Supplement 1 and Figure 3). By treating the degree of age-dependent changes (eg, change from 40 to 70 years) or cross-structure variability as effect sizes, the cross-individual variabilities of volumes (eFigure 2 in Supplement 1) exceeded 10 times the effect size (SD/effect size), whereas those of longitudinal results (Figure 3) were, at most, a few times greater. This finding is particularly important when attempting to evaluate the brain health conditions of individual patients in the future. Many recent studies have shown rapid brain atrophy at the preclinical or early phase of dementia.^32,33,34,35,36^ The data obtained from this study could therefore be a useful resource as a reference to evaluate longitudinal clinical data.

The Alzheimer’s Disease Neuroimaging Initiative (ADNI) is one of the most successful longitudinal studies of brain morphologic characteristics.^37^ Although the ADNI focuses on individuals with cognitive decline and older adults, we examined individuals with normal cognition longitudinally, starting with those in their 30s, to capture the structural changes in middle-aged adults. The UK Biobank is another large imaging study project that covers individuals aged 45 to 69 years and aims to evaluate a healthy population.^38^ However, 1 key strength of our study was the use of serial measurement time points. The UK Biobank includes brain MRI measurements at only 2 time points, with a 2-year interval. Our study design allowed us to capture more detailed information about the structural changes that occur in the brain over time.

Characterizing the normal distributions of brain volume and atrophy rates in a cognitively healthy population can facilitate stratification of high-risk subgroups. We found significant differences in the rate of brain atrophy among individuals, even within the same age group (Figure 1 and Figure 3). Previous research has suggested that populations with significantly enlarged ventricles are at increased risk of developing dementia.^4,12^ Therefore, our brain volume and atrophy rate profile may serve as a useful tool for identifying individuals with nonnormal distributions who are at an increased risk of neurodegenerative diseases. Further research is needed to improve understanding of the genetic, lifestyle, and other modifiable risk factors that may be associated with the differences in brain atrophy rates.

### Limitations

This study has several limitations. First, the cohort was limited to Japanese individuals; therefore, our results may not be generalizable to other races and ethnicities.^39^ Second, relatively few participants were in their 80s, which may have increased measurement variability in this age group. The present study focused on individuals who were considered asymptomatic or cognitively normal, rather than normal or disease-free participants. Although participants were evaluated for cognitive function at baseline and during follow-up visits, molecular imaging, fluid, or genetic biomarkers were not available. Consequently, individuals in the preclinical stage of Alzheimer disease (AD) or AD-related diseases may have been included, which could have contributed to interindividual variability and potentially overestimated the variability observed in this study. Third, the demographic characteristics of the participants were skewed toward wealthier individuals who could afford costly scans or who have health benefits that include MRI. Fourth, due to the limited sample size of female data, we did not perform detailed analysis of sex-related differences, but the preliminary data are available in eTables 2, 4, and 5 in Supplement 1. It is an important future effort to further investigate sex-related differences as more data become available. In addition, because this study did not include patients with cognitive impairment, the discrimination or stratification of groups with normal cognition and cognitive impairment is unavailable. It is of interest to investigate if availability of longitudinal data would improve our ability for diagnosis, especially when the information is combined with multimodal diagnosis of AD and AD-related disease.

## Conclusions

We used serial MRI scans to follow a large sample of asymptomatic adults and characterize age-dependent changes in brain structure volumes and volume change rates. We found characteristic changes in each brain structure, providing a detailed understanding of the spatial and temporal dynamics of normal brain aging and their normal variability. These findings may establish a foundation to deepen our understanding of the aging process and the potential development of age-related neurodegenerative diseases.

## Notes

Supplement 1.
eMethods.
**eFigure 1.** Participant Inclusion and Exclusion Flowchart **eFigure 2.** Ridgeline Plots of Brain Structure Volumes for Each Decade **eFigure 3.** Longitudinal Relationship Between Brain Structure Volumes Over Time Within Different Tissue Types **eFigure 4.** Ridgeline Plots Show the Distribution of Brain Structure Volume-Change Rates for Each Decade **eTable 1.** Sex Distribution Across Different Age Categories **eTable 2.** Longitudinal Relationships Between Brain Structure Volumes Based on Cross-Sectional (Population-Level) and Longitudinal (Individual-Level) Data **eTable 3.** Annual Volume-Change Rates (%/year) and Age Correlations of Brain Structure Volume-Change Rate, Calculated Based on Population- (Cross-Sectional) and Individual-Level (Longitudinal) Data **eTable 4.** Annual Volume-Change Rates (%/year) and Age Correlations of Brain Structure Volume-Change Rate in Women, Calculated Based on Population- (Cross-Sectional) and Individual-Level (Longitudinal) Data **eTable 5.** Annual Volume-Change Rates (%/year) and Age Correlations of Brain Structure Volume-Change Rate in Men, Calculated Based on Population- (Cross-Sectional) and Individual-Level (Longitudinal) Data

Supplement 2.
Data Sharing Statement
