# Supplementary material for: Characterization of Brain Volume Changes in Aging Individuals With Normal Cognition Using Serial Magnetic Resonance Imaging
Source: JAMA Netw Open. 2023 Jun 28;6(6):e2318153. doi: 10.1001/jamanetworkopen.2023.18153 (PMC10308250; doi:10.1001/jamanetworkopen.2023.18153)
Supplement: Supplement 1. — eMethods. eFigure 1. Participant Inclusion and Exclusion Flowchart eFigure 2. Ridgeline Plots of Brain Structure Volumes for Each Decade eFigure 3. Longitudinal Relationship Between Brain Structure Volumes Over Time Within Different Tissue Types eFigure 4. Ridgeline Plots Show the Distribution of Brain Structure Volume-Change Rates for Each Decade eTable 1. Sex Distribution Across Different Age Categories eTable 2. Longitudinal Relationships Between Brain Structure Volumes Based on Cross-Sectional (Population-Level) and Longitudinal (Individual-Level) Data eTable 3. Annual Volume-Change Rates (%/year) and Age Correlations of Brain Structure Volume-Change Rate, Calculated Based on Population- (Cross-Sectional) and Individual-Level (Longitudinal) Data eTable 4. Annual Volume-Change Rates (%/year) and Age Correlations of Brain Structure Volume-Change Rate in Women, Calculated Based on Population- (Cross-Sectional) and Individual-Level (Longitudinal) Data eTable 5. Annual Volume-Change Rates (%/year) and Age Correlations of Brain Structure Volume-Change Rate in Men, Calculated Based on Population- (Cross-Sectional) and Individual-Level (Longitudinal) Data [file jamanetwopen-e2318153-s001.pdf]

Supplementary Online Content

Fujita S, Mori S, Onda K, et al. Characterization of brain volume changes in aging individuals with normal cognition using serial magnetic resonance imaging. *JAMA Netw Open.* 2023;6(6):e2318153.
doi:10.1001/jamanetworkopen.2023.18153

eMethods.
eFigure 1. Participant Inclusion and Exclusion Flowchart
eFigure 2. Ridgeline Plots of Brain Structure Volumes for Each Decade
eFigure 3. Longitudinal Relationship Between Brain Structure Volumes Over Time Within Different Tissue Types
eFigure 4. Ridgeline Plots Show the Distribution of Brain Structure Volume-Change Rates for Each Decade
eTable 1. Sex Distribution Across Different Age Categories
eTable 2. Longitudinal Relationships Between Brain Structure Volumes Based on Cross-Sectional (Population-Level) and Longitudinal (Individual-Level) Data
eTable 3. Annual Volume-Change Rates (%/year) and Age Correlations of Brain Structure Volume-Change Rate, Calculated Based on Population- (Cross-Sectional) and Individual-Level (Longitudinal) Data
eTable 4. Annual Volume-Change Rates (%/year) and Age Correlations of Brain Structure Volume-Change Rate in Women, Calculated Based on Population- (Cross-Sectional) and Individual-Level (Longitudinal) Data
eTable 5. Annual Volume-Change Rates (%/year) and Age Correlations of Brain Structure Volume-Change Rate in Men, Calculated Based on Population- (Cross-Sectional) and Individual-Level (Longitudinal) Data

This supplementary material has been provided by the authors to give readers additional information about their work.

© 2023 Fujita S et al. *JAMA Network Open.*

eMethods

Brain Imaging

From November 2006 to December 2017, high-resolution volumetric fast spoiled gradient-echo was performed with a Signa EXCITE and a Discovery MR750 scanner (repetition time, 6.4 ms; echo time, 2.0 ms; inversion time, 450 ms; field-of-view, 25 cm; flip angle, 15°; acquisition matrix, 256 × 256; number of excitations, 0.5; and voxel dimensions 0.98 × 0.98 × 1.0 mm), using an 8-channel head coil. From January 2018 to April 2021, high-resolution volumetric magnetization-prepared rapid gradient-echo was acquired with a Biograph mMR scanner (repetition time, 1660 ms; echo time, 2.4 ms; inversion time, 910 ms; field-of-view, 25 cm; flip angle, 8°; acquisition matrix, 256 × 256; number of excitations, 1; and voxel dimensions, 0.98 × 0.98 × 1.0 mm) using a 16-channel receiver coil.

© 2023 Fujita S et al. *JAMA Network Open.*

eFigure 1. Participant Inclusion and Exclusion Flowchart

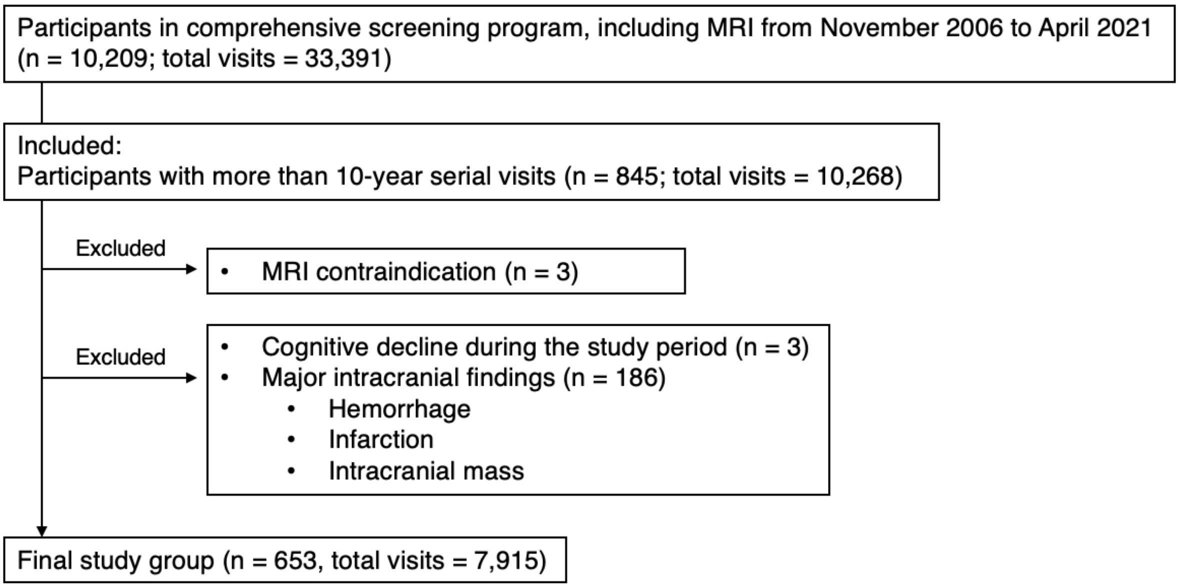

© 2023 Fujita S et al. *JAMA Network Open.*

eFigure 2. Ridgeline Plots of Brain Structure Volumes for Each Decade

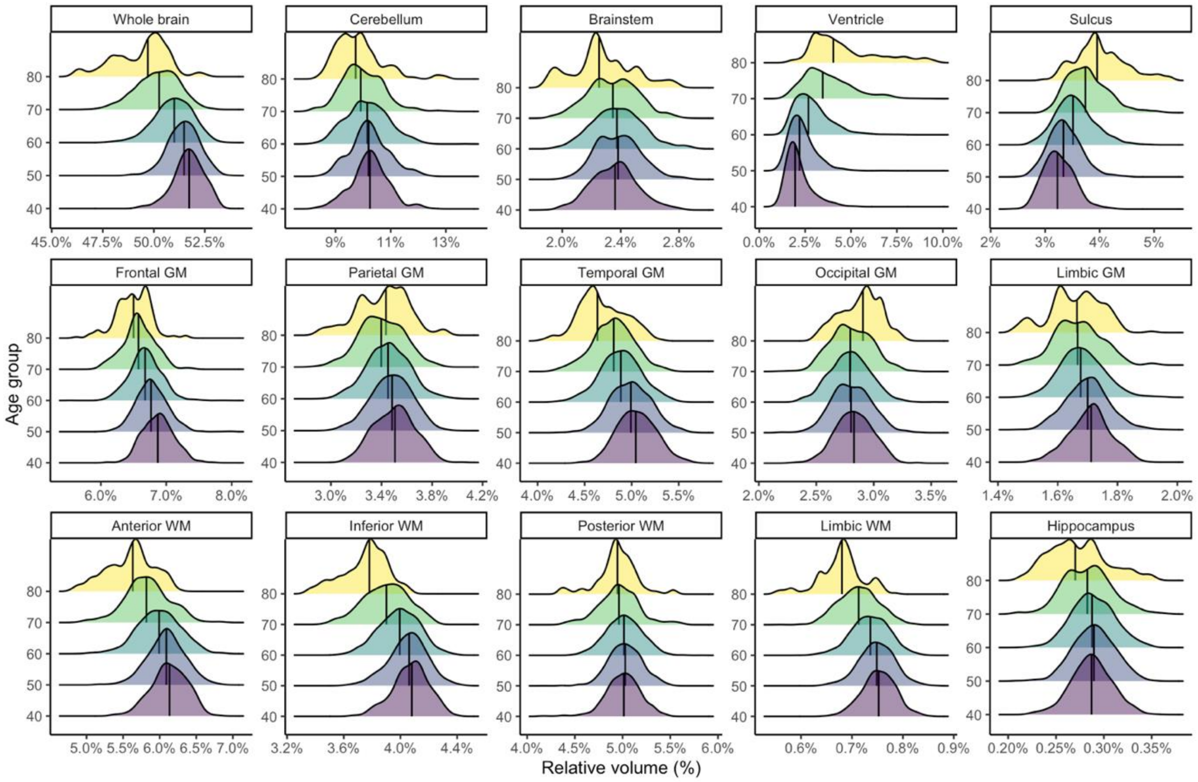

Changes in the distribution of the volume of brain structures over time. The x-axis is the normalized target structure volume (% relative to all structures aggregated). The complicated shape of the distribution in individuals in their 80s is probably due to the small number of observations. GM, gray matter; WM, white matter.

© 2023 Fujita S et al. *JAMA Network Open.*

eFigure 3. Longitudinal Relationship Between Brain Structure Volumes Over Time Within Different Tissue Types

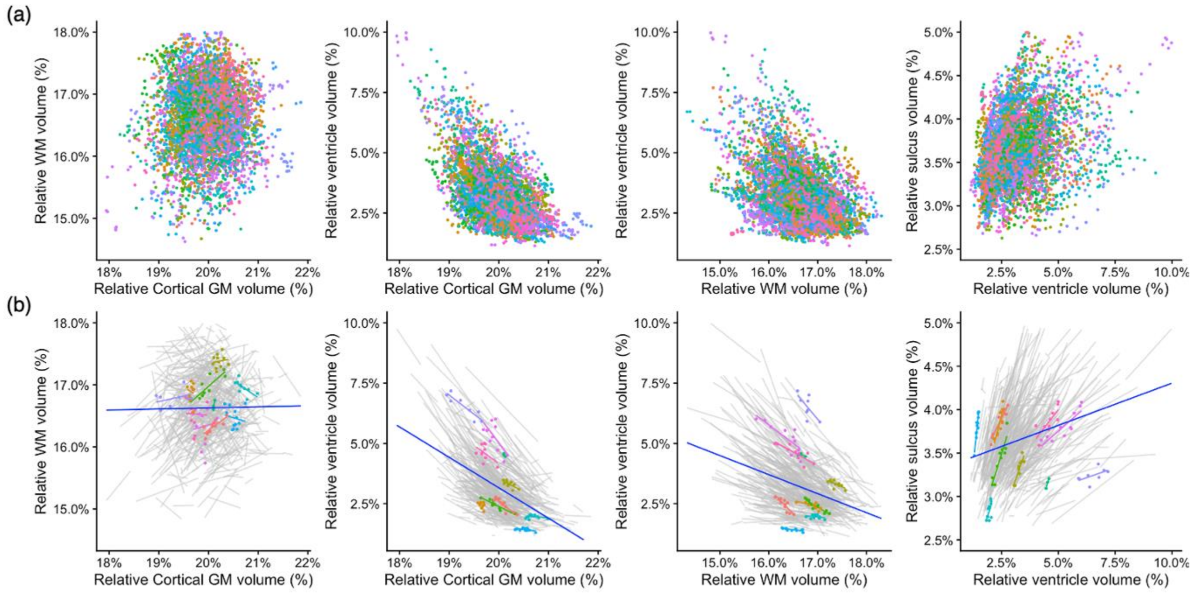

The relationship between brain structure volumes over time within different tissue types is shown in (A) for the whole study population. Each color represents an individual. (B) Best fit lines for each individual (gray lines, individual data), with representative cases visualized using sample points and being color-coded. The blue solid line is the linear fit using data from all visits of all subjects (cross-sectional population data).

© 2023 Fujita S et al. *JAMA Network Open.*

eFigure 4. Ridgeline Plots Show the Distribution of Brain Structure Volume-Change Rates for Each Decade

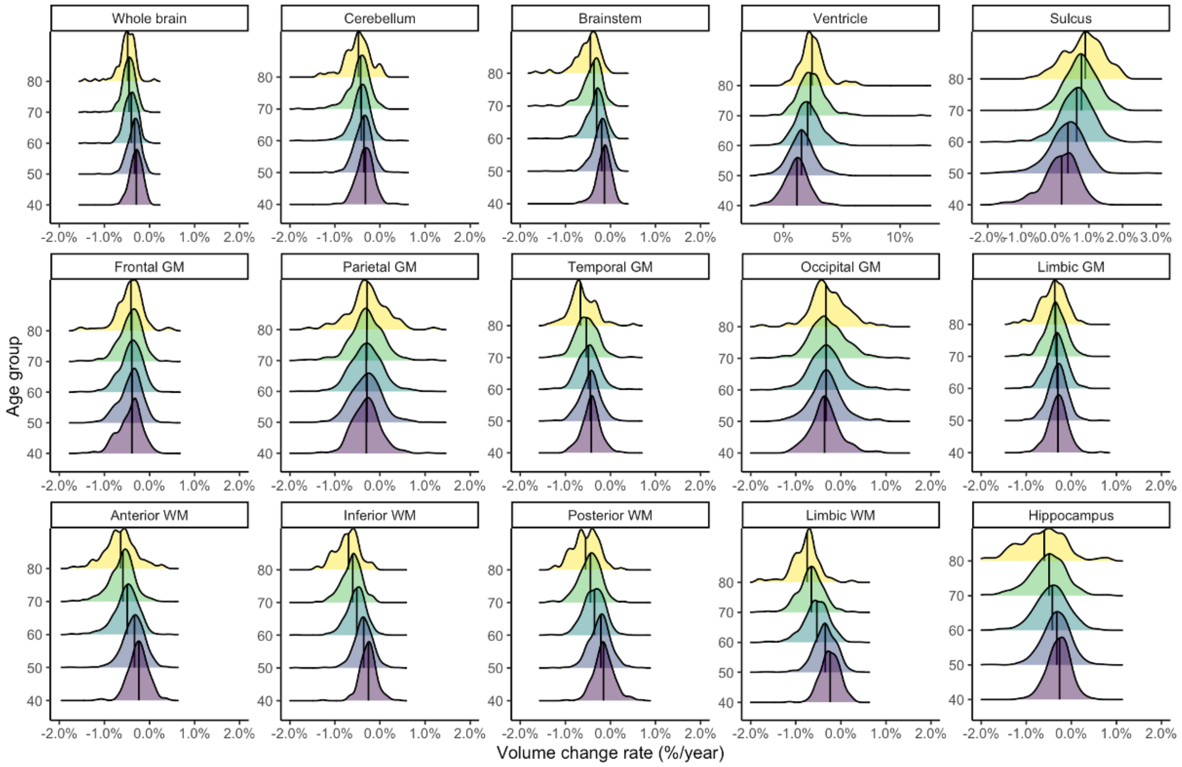

Solid black line represents the median value. The x-axis shows the normalized volume change per year as a percentage of the absolute volume of the given structure. GM, gray matter; WM, white matter.

© 2023 Fujita S et al. *JAMA Network Open.*

eTable 1. Sex Distribution Across Different Age Categories

Age group at visit	Women	Men
30s, ***n*** (%)	3 (8)	33 (92)
40s, ***n*** (%)	324 (26)	945 (74)
50s, ***n*** (%)	735 (28)	1889 (72)
60s, ***n*** (%)	836 (32)	1717 (67)
70s, ***n*** (%)	477 (38)	779 (62)
80s, ***n*** (%)	80 (47)	89 (53)
90s, ***n*** (%)	5 (63)	3 (38)

Note. Categorical data are reported as numerators, and the corresponding percentages are in parentheses.

© 2023 Fujita S et al. *JAMA Network Open.*

eTable 2. Longitudinal Relationships Between Brain Structure Volumes Based on Cross-Sectional (Population-Level) and Longitudinal (Individual-Level) Data

Structural combination	Volume-change correlation (R^2)	
	Cross-sectional	Longitudinal
Cerebral cortex—White matter	0.0002 (0.0067/0.0187)	0.260 [0.072–0.519] (0.361 [0.071–0.768] / 0.397 [0.102–0.755])
Cerebral cortex—Ventricle	0.2905 (0.3470/0.2163)	0.571 [0.221–0.792] (0.608 [0.195–0.889] / 0.603 [0.229–0.887])
White matter—Ventricle	0.1575 (0.1951/0.0485)	0.588 [0.231–0.807] (0.615 [0.223–0.893] / 0.551 [0.179–0.818])
Cerebral cortex—Sulcus	0.0200 (0.0281/0.0083)	0.474 [0.147–0.747] (0.462 [0.095–0.784] / 0.478 [0.118–0.808])
White matter—Sulcus	0.4590 (0.4878/0.3759)	0.658 [0.319–0.855] (0.678 [0.285–0.914] / 0.598 [0.195–0.853])
Ventricle—Sulcus	0.0855 (0.0822/0.0071)	0.736 [0.451–0.868] (0.604 [0.194–0.896] / 0.574 [0.167–0.834])

The R^2 of the volume of the two structures was calculated in a time series for each individual and averaged across individuals to calculate the individual-level correlation. Medians and interquartile ranges are shown for individual-level volume-change correlations. The numbers in the parentheses are from men and women data.

© 2023 Fujita S et al. *JAMA Network Open.*

eTable 3. Annual Volume-Change Rates (%/year) and Age Correlations of Brain Structure Volume-Change Rate, Calculated Based on Population- (Cross-Sectional) and Individual-Level (Longitudinal) Data

Structure	Age correlation (R^2)		Annual volume-change rate (%/year)					
	Cross-sectional	Longitudinal	Overall	40s	50s	60s	70s	80s
Whole brain	0.377	0.728 [0.403–0.875]	-0.390%	-0.300%	-0.342%	-0.414%	-0.460%	-0.508%
Cerebellum	0.019	0.227 [0.083–0.460]	-0.408%	-0.331%	-0.372%	-0.430%	-0.461%	-0.494%
Brain Stem	0.003	0.241 [0.073–0.470]	-0.291%	-0.149%	-0.215%	-0.327%	-0.402%	-0.506%
Cerebral cortex	0.215	0.530 [0.274–0.735]	-0.388%	-0.381%	-0.379%	-0.388%	-0.396%	-0.430%
Frontal GM	0.156	0.368 [0.107–0.662]	-0.423%	-0.438%	-0.418%	-0.416%	-0.426%	-0.446%
Parietal GM	0.035	0.196 [0.057–0.431]	-0.295%	-0.301%	-0.296%	-0.299%	-0.289%	-0.276%
Temporal GM	0.159	0.327 [0.119–0.556]	-0.492%	-0.431%	-0.458%	-0.500%	-0.537%	-0.643%
Occipital GM	0.001	0.214 [0.061–0.461]	-0.315%	-0.340%	-0.330%	-0.314%	-0.286%	-0.273%
Limbic GM	0.036	0.239 [0.070–0.480]	-0.326%	-0.298%	-0.307%	-0.330%	-0.350%	-0.396%
White matter	0.120	0.558 [0.204–0.762]	-0.395%	-0.202%	-0.297%	-0.449%	-0.547%	-0.616%
Anterior WM	0.143	0.387 [0.127–0.630]	-0.454%	-0.246%	-0.349%	-0.517%	-0.620%	-0.649%
Inferior WM	0.178	0.489 [0.153–0.734]	-0.476%	-0.263%	-0.372%	-0.533%	-0.634%	-0.741%
Posterior WM	0.006	0.287 [0.078–0.551]	-0.321%	-0.144%	-0.230%	-0.368%	-0.458%	-0.547%
Limbic WM	0.144	0.325 [0.106–0.563]	-0.479%	-0.237%	-0.354%	-0.541%	-0.670%	-0.794%
Hippocampus	0.008	0.151 [0.035–0.355]	-0.424%	-0.282%	-0.343%	-0.447%	-0.552%	-0.693%
Amygdala	0.020	0.137 [0.029–0.371]	-0.433%	-0.197%	-0.319%	-0.483%	-0.604%	-0.823%
Ventricle	0.280	0.871 [0.625–0.943]	+1.787%	+1.004%	+1.446%	+2.029%	+2.326%	+2.474%
Sulcus	0.255	0.556 [0.229–0.760]	+0.508%	+0.147%	+0.332%	+0.618%	+0.782%	+0.844%

WM, white matter; GM, gray matter
The R^2 of the correlation of age to the given structure was calculated in a time series for each individual and averaged across individuals to calculate the individual-level correlation. Medians and interquartile ranges are shown for individual-level volume-change rate correlations.

© 2023 Fujita S et al. *JAMA Network Open.*

eTable 4. Annual Volume-Change Rates (%/year) and Age Correlations of Brain Structure Volume-Change Rate in Women, Calculated Based on Population- (Cross-Sectional) and Individual-Level (Longitudinal) Data

Structure	Age correlation (R^2)		Annual volume-change rate (%/year)					
	Cross-sectional	Longitudinal	Overall	40s	50s	60s	70s	80s
Whole brain	0.351	0.569 [0.176–0.813]	-0.477%	-0.403%	-0.447%	-0.497%	-0.510%	-0.528%
Cerebellum	0.006	0.241 [0.097–0.476]	-0.535%	-0.443%	-0.497%	-0.553%	-0.585%	-0.620%
Brain Stem	0.001	0.292 [0.063–0.526]	-0.275%	-0.140%	-0.211%	-0.300%	-0.354%	-0.428%
Cerebral cortex	0.333	0.543 [0.239–0.754]	-0.520%	-0.544%	-0.545%	-0.524%	-0.482%	-0.473%
Frontal GM	0.227	0.432 [0.171–0.743]	-0.600%	-0.677%	-0.636%	-0.593%	-0.545%	-0.509%
Parietal GM	0.091	0.196 [0.052–0.485]	-0.427%	-0.475%	-0.475%	-0.444%	-0.355%	-0.253%
Temporal GM	0.229	0.298 [0.095–0.515]	-0.593%	-0.549%	-0.583%	-0.596%	-0.604%	-0.684%
Occipital GM	0.010	0.203 [0.052–0.501]	-0.437%	-0.463%	-0.498%	-0.448%	-0.358%	-0.335%
Limbic GM	0.025	0.243 [0.065–0.475]	-0.446%	-0.434%	-0.435%	-0.443%	-0.455%	-0.517%
White matter	0.051	0.512 [0.194–0.700]	-0.433%	-0.254%	-0.343%	-0.475%	-0.544%	-0.586%
Anterior WM	0.081	0.357 [0.142–0.567]	-0.500%	-0.258%	-0.384%	-0.561%	-0.648%	-0.647%
Inferior WM	0.118	0.377 [0.069–0.703]	-0.506%	-0.321%	-0.416%	-0.543%	-0.618%	-0.685%
Posterior WM	0.001	0.273 [0.078–0.536]	-0.384%	-0.261%	-0.317%	-0.411%	-0.457%	-0.540%
Limbic WM	0.088	0.276 [0.113–0.503]	-0.506%	-0.280%	-0.390%	-0.554%	-0.650%	-0.716%
Hippocampus	0.017	0.135 [0.043–0.342]	-0.515%	-0.311%	-0.396%	-0.520%	-0.664%	-0.944%
Amygdala	0.011	0.125 [0.029–0.380]	-0.451%	-0.229%	-0.327%	-0.469%	-0.596%	-0.893%
Ventricle	0.276	0.778 [0.402–0.917]	+1.515%	+0.317%	+0.980%	+1.792%	+2.157%	+2.587%
Sulcus	0.249	0.425 [0.136–0.679]	+0.300%	-0.214%	+0.016%	+0.419%	+0.6575	+0.690%

WM, white matter; GM, gray matter
The R^2 of the correlation of age to the given structure was calculated in a time series for each individual and averaged across individuals to calculate the individual-level correlation. Medians and interquartile ranges are shown for individual-level volume-change rate correlations.

© 2023 Fujita S et al. *JAMA Network Open.*

eTable 5. Annual Volume-Change Rates (%/year) and Age Correlations of Brain Structure Volume-Change Rate in Men, Calculated Based on Population- (Cross-Sectional) and Individual-Level (Longitudinal) Data

Structure	Age correlation (R^2)		Annual volume-change rate (%/year)					
	Cross-sectional	Longitudinal	Overall	40s	50s	60s	70s	80s
Whole brain	0.440	0.758 [0.504–0.891]	-0.353%	-0.266%	-0.303%	-0.376%	-0.435%	-0.498%
Cerebellum	0.040	0.222 [0.079–0.454]	-0.354%	-0.295%	-0.325%	-0.374%	-0.398%	-0.437%
Brain Stem	0.010	0.217 [0.075–0.449]	-0.298%	-0.152%	-0.217%	-0.339%	-0.427%	-0.541%
Cerebral cortex	0.182	0.519 [0.289–0.723]	-0.332%	-0.327%	-0.317%	-0.326%	-0.352%	-0.410%
Frontal GM	0.143	0.322 [0.076–0.614]	-0.349%	-0.359%	-0.337%	-0.335%	-0.365%	-0.418%
Parietal GM	0.021	0.196 [0.058–0.417]	-0.240%	-0.244%	-0.229%	-0.232%	-0.255%	-0.286%
Temporal GM	0.124	0.345 [0.142–0.565]	-0.449%	-0.392%	-0.412%	-0.456%	-0.502%	-0.624%
Occipital GM	0.001	0.221 [0.066–0.447]	-0.263%	-0.300%	-0.267%	-0.252%	-0.250%	-0.244%
Limbic GM	0.044	0.239 [0.070–0.488]	-0.275%	-0.253%	-0.259%	-0.278%	-0.297%	-0.341%
White matter	0.161	0.592 [0.227–0.788]	-0.379%	-0.184%	-0.279%	-0.438%	-0.549%	-0.629%
Anterior WM	0.180	0.409 [0.118–0.659]	-0.435%	-0.242%	-0.336%	-0.497%	-0.606%	-0.649%
Inferior WM	0.205	0.516 [0.191–0.742]	-0.463%	-0.244%	-0.355%	-0.528%	-0.643%	-0.766%
Posterior WM	0.011	0.291 [0.079–0.556]	-0.294%	-0.106%	-0.197%	-0.348%	-0.459%	-0.550%
Limbic WM	0.186	0.348 [0.102–0.573]	-0.467%	-0.223%	-0.340%	-0.535%	-0.680%	-0.831%
Hippocampus	0.007	0.153 [0.033–0.361]	-0.386%	-0.272%	-0.323%	-0.413%	-0.494%	-0.578%
Amygdala	0.022	0.141 [0.030–0.368]	-0.425%	-0.186%	-0.316%	-0.490%	-0.608%	-0.791%
Ventricle	0.323	0.889 [0.715–0.947]	+1.901%	+1.229%	+1.620%	+2.137%	+2.413%	+2.422%
Sulcus	0.311	0.602 [0.275–0.779]	+0.596%	+0.265%	+0.450%	+0.709%	+0.847%	+0.914%

WM, white matter; GM, gray matter
The R^2 of the correlation of age to the given structure was calculated in a time series for each individual and averaged across individuals to calculate the individual-level correlation. Medians and interquartile ranges are shown for individual-level volume-change rate correlations.

© 2023 Fujita S et al. *JAMA Network Open.*
