# Supplementary material for: Characterization of Brain Volume Changes in Aging Individuals With Normal Cognition Using Serial Magnetic Resonance Imaging
Source: JAMA Netw Open. 2023 Jun 28;6(6):e2318153. doi: 10.1001/jamanetworkopen.2023.18153 (PMC10308250; doi:10.1001/jamanetworkopen.2023.18153)
Supplement: Supplement 2. — Data Sharing Statement [file jamanetwopen-e2318153-s002.pdf]

Data Sharing Statement

Fujita. Characterization of Brain Volume Changes in Aging Individuals With Normal Cognition Using Serial Magnetic Resonance Imaging. *JAMA Netw Open*. Published June 28, 2023. doi:10.1001/jamanetworkopen.2023.18153

Data
Data available: Yes
Data types: Data dictionary
How to access data: Data generated or analyzed during the study can be requested from the principal investigator, given appropriate ethical and data protection approvals and data transfer agreements.
When available: With publication

Supporting Documents
Document types: None

Additional Information
Who can access the data: Data generated or analyzed during the study can be requested from the principal investigator, given appropriate ethical and data protection approvals and data transfer agreements.
Types of analyses: None.
Mechanisms of data availability: Data generated or analyzed during the study can be requested from the principal investigator, given appropriate ethical and data protection approvals and data transfer agreements.
